# Social networks of patients with psychosis: a systematic review

**DOI:** 10.1186/s13104-015-1528-7

**Published:** 2015-10-12

**Authors:** Claudia Palumbo, Umberto Volpe, Aleksandra Matanov, Stefan Priebe, Domenico Giacco

**Affiliations:** Unit for Social and Community Psychiatry, Newham Centre for Mental Health, Queen Mary University of London, London, E13 8SP UK; Department of Neuroscience, University of Bari, Bari, Italy; Department of Psychiatry, Second University of Naples, Naples, Italy

**Keywords:** Schizophrenia, Psychosis, Social network, Friendship, Social relationships

## Abstract

**Background:**

Social networks are important for mental health outcomes as they can mobilise resources and help individuals to cope with social stressors. Individuals with psychosis may have specific difficulties in establishing and maintaining social relationships which impacts on their well-being and quality of life. There has been a growing interest in developing social network interventions for patients with psychotic disorders. A systematic literature review was conducted to investigate the size of social networks of patients with psychotic disorders, as well as their friendship networks.

**Methods:**

A systematic electronic search was carried out in MEDLINE, EMBASE and PsychINFO databases using a combination of search terms relating to ‘social network’, ‘friendship’ and ‘psychotic disorder’.

**Results:**

The search identified 23 relevant papers. Out of them, 20 reported patient social network size. Four papers reported the mean number of friends in addition to whole network size, while three further papers focused exclusively on the number of friends. Findings varied substantially across the studies, with a weighted mean size of 11.7 individuals for whole social networks and 3.4 individuals for friendship networks. On average, 43.1 % of the whole social network was composed of family members, while friends accounted for 26.5 %.

**Conclusions:**

Studies assessing whole social network size and friendship networks of people with psychosis are difficult to compare as different concepts and methods of assessment were applied. The extent of the overlap between different social roles assessed in the networks was not always clear. Greater conceptual and methodological clarity is needed in order to help the development of effective strategies to increase social resources of patients with psychosis.

**Electronic supplementary material:**

The online version of this article (doi:10.1186/s13104-015-1528-7) contains supplementary material, which is available to authorized users.

## Background

The term “social network” (SN) originated in sociology and social anthropology and describes a set of significant relationships of an individual. Research has highlighted the importance of social networks for both mental and physical health. Social relationships help individuals to cope with social stressors and improve their quality of life [1]. In large cohort studies social isolation has been found to be a major risk factor for morbidity and mortality [2, 3].

Social networks play an important role in patients with psychosis as they can mobilise resources, provide information, and help patients manage their illness [4]. The characteristics of patients’ networks influence their well-being, social functioning and use of mental health services [4–6]. Individuals with psychosis frequently experience difficulties in developing and maintaining social relationships [7]. Their social networks tend to be smaller than those of non-clinical populations, and are mainly composed of family members [5, 8]. Social withdrawal seems to start early as the reduction in network size often pre-dates the onset of the psychotic symptoms [9]. This is possibly due to associated neurocognitive deficits that impact on social functioning [10]. A role could also be played by childhood (or later) stressors that may result in difficulties in social interactions and in the individual becoming socially isolated [11]. On the other hand, social withdrawal may be seen as a protective mechanism for reducing arousal and preventing relapse in patients who find communication overwhelming [12, 13]. The type of symptoms, length of illness and frequency of hospitalisation may also impact on the number and quality of patients’ social ties [10, 14, 15]. Stigma attached to a diagnosis of schizophrenia and related disorders can significantly reduce opportunities to form relationships. Social disadvantage resulting from loss of employment and financial problems may push patients further into social isolation.

Some authors have suggested that changes in modern societies may reinforce social deficits in people with psychosis [16]. The ability to form new social relations outside the family circle is becoming increasingly important in contemporary society [17, 18], as a consequence of changes in family structure and of the increased number of people living alone [19]. Friendships as voluntary associations need time to develop and effort is required to negotiate the issues of mutual trust, intimacy and commitment [10, 13]. The size of friendship networks may be considered as an indicator of patients’ ability to establish relationships outside a given set of family relations.

Social network size appears important for patients with psychotic disorders, both as a relevant outcome criterion of interventions in its own right and as a factor influencing other types of outcomes such as quality of life and service use. Assessing in a systematic manner social networks of people with severe mental illness has recently been advocated as a priority for mental health research [20].

Against this background, the aim of this study is to systematically review the papers reporting the size and composition of social networks as well as the size of friendship networks in patients with psychosis. This will provide information on social needs of this population as well as an insight on advantages and limitation of current assessment methods. Such information may help the development of social interventions tailored on the needs of people with psychosis.

## Methods

### Search strategy and selection criteria

The review process was conducted according to the protocol developed. The literature search was completed on 07th September 2013, and updated on June 16th 2015.

Primary research papers relevant to the review were identified using online searches on health information for London online system (HILO) with the MEDLINE, EMBASE and PSYCINFO databases.

Searches were conducted using Boolean ‘AND/OR’ operators and wildcards as appropriate with a combination of terms pertaining to social networks with terms used to designate a cluster of diagnoses.

The following search terms were employed:([“social network” or “social contact” or friendship) and (“schizophrenia” or “psychosis” or “psychotic disorder”) in title and abstract. We also carried out a hand search for studies in key-journals, reviews on the topic and conference abstracts. The titles and abstracts of all identified papers were reviewed to assess their relevance. An independent researcher (CH) was allocated a random selection (20 %) of abstracts for screening to determine inclusion, using a web-based random integer generator [21]. Based on this evaluation, all potentially relevant articles were retrieved and exported into a reference citation manager.

The reference lists of identified papers or texts were systematically scrutinized in order to identify any literature not present in electronic databases. Newly found abstracts were examined to determine their relevance. Authors were contacted in cases where required data were not reported, to clarify some concepts or for further information. Duplicates were manually removed.

We recorded the data on the number of potential papers identified; papers assessed for eligibility, papers excluded, eligible papers, and finally the number of papers included in systematic review.

### Inclusion and exclusion criteria

In a second stage of the search, studies were included if they were conducted on adults (i.e. ≥18 years of age) suffering from a psychotic disorder (i.e. including a standardized diagnosis of either schizophrenia, schizoaffective disorder, “narrow schizophrenia” spectrum disorder, or “psychosis”).

Papers were excluded if social networks were not explicitly assessed and described, for example: papers exploring the broader social functioning with no specific reference to social networks; papers on deficits in social cognition and other neuropsychological functions; and papers not reporting primary data. Reviews have been excluded, but their reference list has been explored in detail in order to find relevant papers not captured through the main search strategy. Following that, we excluded all literature that did not report actual data on the size of social networks of people with psychotic disorders. Two further studies were excluded as they did not report mean size of social networks [22, 23]. There were no restrictions on study design, publication year or language.

### Data extraction

A data extraction form was developed and piloted for particular questions to be addressed by the review and included study characteristics (study design, study setting, study eligibility criteria, aims, methods, and research design), patients characteristics, instruments used, and network size.

The screening of the papers was performed according to the preferred reporting items for systematic reviews and meta-analyses (PRISMA) flow diagram [24] and the checklist.

Data were extracted by one reviewer (CP) and checked by a second reviewer (UV). Divergent interpretations were resolved through discussion between them and with a third reviewer (AM). Two further researchers (DG and SP) were available for further discussion.

### Statistical analyses

Available data on the size of social network and number of friends were summarized as weighted arithmetic means to account for differences in sample sizes across the included studies. Initially, we aimed to perform meta-analysis but this was not possible due to the heterogeneity of social network definitions and methodologies used in the studies.

## Results

### Selection of studies

**Fig. 1:**
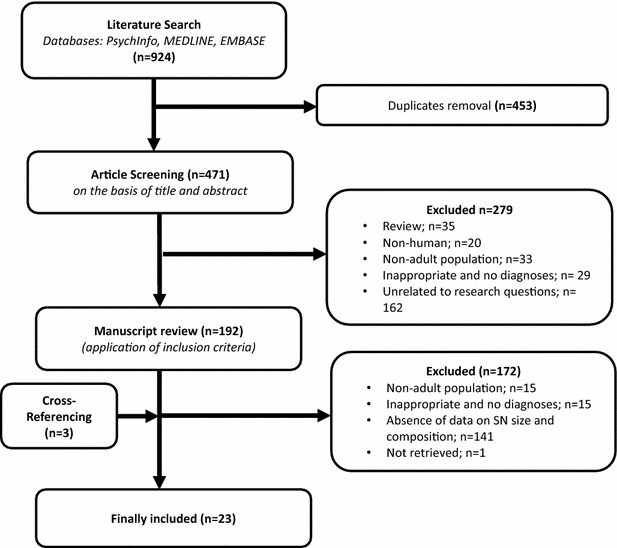
PRISMA diagram, showing the literature search process

A total of 924 records were retrieved. After the removal of duplicates and the application of inclusion and exclusion criteria, 192 publications remained. Twenty-three studies included in the review reported data on the mean size of social networks, including networks of friends. The included papers were published between 1976 and 2013. The details of the selection procedure are displayed in Fig. 1. The PRISMA checklist for this review is reported in Additional file 1.

### Characteristics of studies

Out of 23 papers selected for the review 20 reported the mean size of multi-category social networks. In addition to the size of whole networks, four out of these 20 papers also reported the mean number of friends. A further three papers reported the data on the mean size of friendship networks only. The characteristics of studies included in the review are reported in Table 1.

**Table 1:** Characteristics of studies assessing the size of social networks including friendship networks

No.	Study description authors’ name, year, country	Sample with psychosis	Diagnostic criteria	Participants and Setting	Social network size assessment	Minimum frequency of contact and/or timescale	Type of social relationships considered	Network components reported
1	Angermeyer and Klusmann, 1987, Germany [55]	30	RDC	Recently discharged patients in community	Interview schedule for the assessment of social relationships	Once in the past week	–	Family, other patients and professional helpers
2	Becker et al., 1998, United Kingdom (UK) [47]	129	ICD-10	Patients in community	Social network achedule, SNS [35]	Once in the previous month	All relationships	Relatives, friends, and other contacts^a^
3	Cohen and Sokolovsky, 1978, United States (USA) [31]	32	Clinical assessment	Patients in community residences	Semi-structured “Network Profile Questionnaire”	Once per month in a preceeding 12 months	Exclusively formal contacts excluded (i.e.with psychiatrists and social workers)	Kin and non-kin
4	Cohen and Kochanowicz, 1989, USA [52]	47	DSM-III	Psychiatric clinic outpatients	Modified network analysis profile, NAP [30, 31]	Once in previous 3 month once in 12 months for important persons	All relationships	Kin, non-kin and formal sector
5	Cohen et al., 1996, USA [64]	117	DSM-III-R	Patients in community residences and psychiatric clinic outpatients	Network analysis profile, NAP [30, 31]	Notable interaction^b^ within the past 3 months for non-kin; within 12 months for kin	All relationships	Formal and informal sector^c^
6	Dozier et al., 1987, USA [65]	18	DSM-III	Outpatients of the intervention programme for young adults	Study specific semi-structured questionnaire	Once in the past 2 weeks	All important persons except hospital staff and other patients	–
7	Estroff et al., 1994, USA [48]	81	N/A	Inpatients and patients in community	Study specific semi-structured questionnaire	–	All relationships	Relatives, friends and mental health professionals
8	Famiyuwa and Olatokunbo, 1984, Nigeria [43]	85	Feighner criteria	Outpatients of community based counseling service	Study specific semi-structured questionnaire	Twice per week	On-going relationships with family and co-workers	Family and co-workers
9	Hamilton et al., 1989, USA [56]	39	DSM-III	Outpatients of mental health clinic for veterans	Modified Pattison psychosocial kinship inventory, PPKI [36, 37]	–	Subjectivelly important relationships	Kin and non-kin
10	Harley et al., 2012, UK [13]	137	DSM-IV	Patients in community	Study specific semi-structured questionnaire	Once per week over the past 3 months	Friends defined as non kin, non-services providers with evidence of shared interests and activities	Friends only
11	Hernando et al., 2002, Spain [58]	32	DSM-III-R	Outpatients in mental health day centres	Study specific semi-structured questionnaire	–	All relationships	Professionals and others^d^
12	Horan et al., 2006, USA [54]	89	DSM-III-R and RDC	Inpatients	Study specific semi-structured questionnaire based on Hammer [41] and Randolph [42]	12 months	Frequent or important relationships except those with treatment providers and persons of less than 10 years of age Patient is in conatc or close to,but less than 10-year-old and treatment providers	Kin
13	Kauranen et al., 2000, Finland [50]	29	DSM-III-R	Inpatients and outpatients	Klefbeck’s social network map [38]	_	All current relationships	Family, friends, co-workers and formal sector
14	Lipton et al., 1981, USA [45]	30	RDC	Inpatients	Modified network analysis profile, NAP [30, 31]	Once in the past year	All relationships	Kin, non –kin and formal sector
15	Meeks and Hammond, 2001, USA [57]	120	RDC	Patients in community	Modified network analysis profile, NAP [30, 31]	Once in the past 3 months for non-kin; once per year for kin and service providers if important	Persons known by name in kin, non-kin and service sector	Kin, non-kin and service sector
17	Pessoa Moreno Macedo et al., 2013, Brazil [49]	17	ICD-10	Outpatients in community based services	Semi-structured questionnaires based on Sluzki [40]	–	–	Family, friends, community and work/study sector
16	Pernice-Duca, 2008, USA [44]	103	DSM-IV	Patients in community participating in clubhouse programmes	Social network analysis approach by McCallister and Fischer [32, 33]	–	Important relationships	Family, friends, professionals, clubhouse staff and peers
18	Sawicka et al., 2013, Poland [46]	105	ICD-10	Patients in community using home care services	The map and the questionnaire of social support [39]	–	Individuals with whom patient is in contact	Persons in the same household, closest family, other relatives, colleagues, neighbours, other acquaintances, therapists, and other persons
19	Seidmann et al., 1987, USA [53]	15	DSM-III	2 years after 1st psychiatric hospitalisations	Modified network analysis profile, NAP [30, 31]	Once in the past 3 months	All individuals known by name	–
20	Sibitz et al., 2010, Austria [59]	157	ICD-10	Inpatients and day clinic care outpatients	A question about the number of friends	–	Friends based on self-defintion	Friends only
21	Thorup et al., 2007, Denmark [51]	578	ICD-10	Inpatients and outpatients	Social network schedule, SNS [35]	Once in the past month	Friends based on self-defintion	Friends only
22	Tolsdorf, 1976, USA [66]	10	N/A	Inpatients	Study specific semi-structured questionnaire	–	–	Kin
23	Van Humbeeck et al., 2000, Belgium [67]	56	DSM-IV	Patients in supported living community residences	Modified social network scale by McCallister and Fischer [32, 33] and Fischer [34]	–	Important formal and informal persons	–

^a^Other contacts: nonfriends such as acquaintances, shopkeepers, health or social or other service staff

^b^Examples of notable interaction: a 15 min conversation; a material exchange such as goods or money, or social outing

^c^Informal sector: kin, friends and acquaintances

^d^Friends, acquintances, neighbours

Twelve out of 23 studies were conducted in the USA (N = 757 participants) and nine in Europe (N = 1253).European studies were carried out in Austria (N = 157), Belgium (N = 56), Denmark (N = 578), Finland (N = 29), Germany (N = 30), Poland (N = 105), Spain (N = 32) and two in the United Kingdom (N = 266). One study was carried out in Africa (Nigeria, N = 85) and one in South America (Brazil, N = 17).

The whole social networks were assessed for a total of 1184 patients, and friendship networks for a total of 1163 patients.

Thirteen studies assessed participants diagnosed with schizophrenia only, while four studies also included patients with schizoaffective or schizophreniform disorder. Patients with schizophrenia spectrum disorders were assessed in six studies. With regard to diagnostic criteria, seven studies used DSM-III and DSM-III-R [25], while further three used DSM-IV [26]. Five studies utilised ICD-10 [27], three RDC [28] and one the criteria according to Feigner et al. [29]. In one study participants were diagnosed using either DSM-III-R or RDC criteria. Three papers did not specify which diagnostic criteria were used.

In terms of study setting, fourteen studies assessed patients living in the community including outpatients of mental health clinics and those who attend day centres. Two studies reported findings on the networks of currently hospitalised patients, while a further three specifically followed up patients that were previously hospitalised. Four papers reported that their samples included both inpatients and outpatients.

### Assessment of social networks and friendship networks

Social networks were conceptualised in different ways in the papers included in the review, depending on the aims of the study and measures applied. The assessment approaches used, including the frequency of contacts and timeframes observed for the inclusion in the network, are reported in Table 1.

Six studies used the network analysis profile approach (NAP) [30], originally developed by Cohen and Sokolovsky [31] to assess social networks. Two studies based their assessments on the modified social network analysis approach by McCallister and Fischer [32, 33] and Fischer [34]. In addition to these approaches, the social network schedule (SNS) [35]; Pattison psychosocial kinship inventory (PPKI) [36, 37]; Klefbeck’s social network map [38]; the map and the questionnaire of social support (including the map of social network) [39]; questionnaires ‘Gerador de Nomes and Qualificador do Apoio Social’ e ‘Gerador de Atributos para oVínculo’ based on Sluzki [40] and a semi-structured questionnaire based on Hammer [41] and Randolph [42] were applied in one study each. Six papers described using semi-structured questionnaires developed specifically for the studies in question. A further three papers that focused on friendship networks used the following instruments: social network schedule approach [35]; a semi-structured questionnaire developed for the study; and a question about the number of friends/people patients feel close to.

Depending on the assessment approaches used, different frequencies of contacts occurring over varying lengths of time were considered to establish whether an individual belongs to a social network of a patient or not. The stipulated frequency of contacts varied from twice per week to at least once per year. Three studies allowed less frequent contact for kin or “important” persons in the network. Ten out of 23 papers did not specify frequency of contact and/or timeframe used. Seven papers that reported the size of friendship networks also used different frequencies of contact and different timescales, from current contacts to those occurring at least once per year, while two did not specify these parameters.

Most studies that investigated multi-category social networks mapped either all the patient’s contacts occurring within the observed time or those regarded as subjectively important. Those relationships that met the specified minimum contact frequency and/or other parameters were later categorised according to the social roles assumed in the patient’s network. Three studies excluded treatment providers such as psychiatrists and social workers from network membership, as their focus was on measuring support outside the treatment setting. Several studies used more restricted definitions of social network, for example, focusing on family members and co-workers only [43].

Seven studies that reported the size of friendship networks mostly relied on self-definition of friendship. The extent of the overlap between friends and other social figures such as kin and mental health professionals was not always clear. In response to this, Harley et al. [13] defined friends as individuals in the network who were non-kin; not part of service provider system; with evidence of shared activities, interests and interaction; and with actual contact occurring at least once in the past 3 months. In addition, the studies included did not always distinguish between friends and acquaintances. For example, Pernice-Duce [44] stated that friends consisted of general friendships, acquaintances in the community, roommates, neighbours and co-workers.

### Social network size

The findings on the mean size of whole multi-category social networks were reported by 20 studies. The figures are presented in Table 2.

**Table 2:** Mean size of social networks and their composition

Study description		Network size (mean)		Network composition (%)^a^					
No.	Authors’ name, year	Whole network	Friendship network	Kin	Friends	Non-kin	Other patients	MH and other professionals	Others social figures
1	Angermeyer and Klusmann, 1987 [55]	11.8	–	38.9			8	8.5	
2	Becker et al., 1998 [47]	12.8	4.3	30	33				32^b^
3	Cohen and Sokolovsky, 1978 [31]	13.3	–						
4	Cohen and Kochanowicz, 1989 [52]	7.7	–	46.6		32.5		20.9	
5	Cohen et al., 1996 [64]	10	–						
6	Dozier et al., 1987 [65]	16.3	–						
7	Estroff et al., 1994 [48]	11.4	–	68.7	24.4			6.9	
8	Famiyuwa and Olatokunbo, 1984 [43]	11.6	–	52					48^c^
9	Hamilton et al., 1989 [56]	12.9	–	48.1		52			
10	Harley et al., 2012 [13]	–	1.6						
11	Hernando et al., 2002 [58]	11.8	–						
12	Horan et al., 2006 [54]	8.8	–	64					
13	Kauranen et al., 2000 [50]	18.6	5	65.1	27			0.5	6.5^d^
14	Lipton et al., 1981 [45]	10.9	4.7	48.6	42.6			9.2	
15	Meeks and Hammond, 2001 [57]	18.7	–	53		35		10	
16	Pessoa Moreno Macedo et al., 2013 [49]	10.1	–	68.6	15.7	13.4			2.3
17	Pernice-Duca, 2008 [44]	4.6	1	34.5	21.6		11.6	32.3	
18	Sawicka et al., 2013 [46]	6.0	–						
19	Seidmann et al., 1987 [53]	44.9	–						
20	Sibitz et al., 2010 [59]	–	4.6						
21	Thorup et al., 2007 [51]	–	3.6						
22	Tolsdorf, 1976 [66]	29.8	–	61.1					
23	Van Humbeeck et al., 2000, Belgium [67]	11.6	–						

^a^For some studies, we do not have information on all the types of contacts and the percentages do not add up to 100 %. We reported them in an effort to provide as much information as possible

^b^Other contacts: nonfriends such as acquaintances, shopkeepers, health or social or other service staff

^c,d^Co-workers

There was a substantial variability in values with figures ranging from 4.6 to 44.9, reflecting the variety of network concepts and assessment approaches used. The weighted mean of the social network size for patients included in these studies (N = 1184) was 11.7.

Lipton et al. [45] found that first admission patients with schizophrenia had larger social networks (15.5) than those with multiple admissions (6.3). Patients with psychosis who lived in the community had significantly smaller social networks than individuals without psychiatric history [31]. Sawicka et al. [46] did not find a significant difference in size of social networks for male and female patients with schizophrenia (5.83 vs. 6.15, t = −0.416; p = n.s).

### Social network composition

Social networks of patients encompassed a variety of social figures: family/kin (blood and marital ties), non-kin (friends, acquaintances, neighbours), co-workers, other patients and different professionals (mental health and other medical professionals, social workers, carers, teachers). Fourteen out of 20 studies reported the mean size or percentage of specific network segments. The figures are presented in Table 2.

The percentage of family/kin members in the networks ranged between 30 % [47] and 68.7 % [48], and friends between 15.7 % [49] and 42.6 % [45]. The weighted percentage of kin across these studies was 43.1 % and friends 26.5 %.

### Friendship network size

The findings on the mean size of friendship networks were reported by seven studies. The figures are displayed in Table 2. The mean values varied between 1 [44] and 5 [50]. The weighted mean of the friendship network size for patients included in these studies (N = 1163) was 3.4.

A greater mean number of friends was found for female (4.1) than for male patients with psychosis (3.3), assessed in both in- and outpatient settings [51].

### Associations with social network and friendship size

Cohen and Kochanowicz [52] reported a negative association between age and social network size (z = −0.24, p < 0.05), however this was not confirmed by the findings of Seidman et al. [53] who found no significant link. One study reported larger social networks in married patients [47]. No significant associations were found between SN size and age of onset and length of prodromal period, [54], or illness duration [54, 55]. Higher levels of negative symptoms were associated with smaller SNs (π = −0.64, p < 0.001) [56]; (p = 0.002) [57]; (β = −0.18, p = 0.002) [58]. A longitudinal study of first episode patients with schizophrenia found no significant correlation between network size and BPRS scores at baseline, however, at 1 year follow up, scores on thought disturbance scale significantly correlated with smaller network size (r = −0.36, p < 0.05) [54]. Poor social network contributed to a lack of empowerment and greater stigma, but had no direct effect on quality of life [59].

## Discussion

### Main results

The findings on the mean size of social networks in patients with psychosis, including friendship networks were reported in 23 papers. The networks with more than one category of social figures were assessed in 20 studies for a total of 1184 patients. The number of friends was reported for a total 1163 patients in seven studies.

Social networks and friendship have been conceptualized in different ways and a variety of approaches were used to assess them. Methodological inconsistencies have resulted in limited comparability of the results.

In our study, patients with psychosis had on average 11.7 individuals in their social networks, while the average number of friends was 3.4. These figures varied substantially across studies, i.e. for whole social network size figures reported ranged from 4.6 to 44.9. The social networks were family-dominated with on average 43.1 % of network members being relatives, in contrast to 26.5 % of members categorised as friends.

With regard to patients’ characteristics associated with network size, the higher levels of negative symptoms and not being married may be associated with smaller networks [56–58]. There have been mixed findings on the relationship between age and network size [52, 53].

### Strengths and limitations

This paper has systematically reviewed the evidence on the size of social networks of patients with psychosis, as well as the size of friendship networks. The search strategy allowed to capture a large number of studies. Different researchers independently extracted and reviewed the data. No language restrictions were adopted (four non-English papers were included) and when necessary the authors were contacted to clarify ambiguous information.

However, a number of limitations must be considered while interpreting the results of this study.

The number of studies containing data on the size of social networks was relatively small. Almost half of the studies were carried out in the United States which may have influenced the findings.

The heterogeneity of methodological approaches used to study social networks warrants caution when interpreting the findings. Social networks were conceptualised and assessed in different ways. The criteria for inclusion in social network varied across studies both in terms of frequency of contacts and timeframes considered. Some studies assessed all the contacts while others focused on specific categories. There were no algorithms for deriving scores from one study to another thus hindering the possibility to perform a meta-analysis of the findings on network size. A limited number of included studies reported the composition of social networks.

The definition of a “friend” and the extent of the overlap with other social roles was not clear in most studies as they relied on self-definition of friendship. The numbers of friends reported in our study varied substantially depending on the definition of friendship used.

A further limitation is that only one study [31] compared SN size of people with psychosis with those of people who did not have an established psychiatric diagnosis. Moreover, that study was carried out in people who were hosted in a hostel because of social disadvantage, therefore the results of this comparison may not as such be applicable to the general population.

### Comparison with the literature

Our findings are comparable to the average network size of 13 reported by both a non-systematic review on networks in patients with psychosis [8] and a systematic review on those suffering from severe mental illness [5]. Some authors have argued that the real size of networks reported by people with severe mental illness may be even smaller, questioning their ability to accurately perceive and evaluate their social resources [60].

Social networks of patients with psychosis assessed in our study were family-dominated with, on average, 43.1 % of network members drawn from relatives. Only 26.5 % of network members were described as friends. This is line with other findings on people with severe mental illness [5]. In contrast, family and friends contributed equally to the overall social network of patients with HIV/AIDS [61].

With regard to the size of friendship networks, the average number of 3.4 friends found in our review was much lower than the figures reported for the general population in the UK (N = 10.6 for men and N = 7.6 for women) [62]. More than half of people with severe mental illness reports problems with loneliness which may be linked to small friendship networks [63]. Friends as a source of support are becoming increasingly important in contemporary society due to increased mobility and the growing number of people living alone [10]. The presence of friends may provide emotional and practical support to patients with a psychological disorder and help them to cope with life stressors. Friends may also help them to preserve self-identity and their sense of worth in challenging circumstances [1]. Patients with psychosis with friends-dominated or friends-inclusive social networks were found to have less difficulties in self-care than those with family-dominated networks [6].

## Conclusions

The findings of this review suggest a number of avenues for further research.

The studies that were included showed significant conceptual and methodological heterogeneity which limited the comparability of their results. Comprehensive and conceptually-driven methods and assessment tools are needed to assess social relations of people with psychosis.

In-depth explorations of what are the specific difficulties of people with schizophrenia in establishing or maintaining social contacts and how their social relationships differ from unaffected controls should be carried out.

These are required steps to allow the development of effective strategies to increase social support for people with psychosis and to be able to test their effectiveness.

It may also be worth noting that many of the examined studies were conducted in times when the internet and social media were not part of our daily interactions, and future research may benefit from exploring the virtual networks of people with psychotic disorders.

## Additional file

10.1186/s13104-015-1528-7 PRISMA checklist.

## Abbreviations

SN: social network
SNs: social networks
NAP: network analysis Profile
SNS: social network schedule
PPKI: Pattison psychosocial kinship inventory
HILO: health information for London online system
PRISMA: preferred reporting items for systematic reviews and meta-analyses
